# Synthesis of centimeter-size free-standing perovskite nanosheets from single-crystal lead bromide for optoelectronic devices

**DOI:** 10.1038/s41598-019-47902-1

**Published:** 2019-08-13

**Authors:** Jian-Yao Zheng, Hugh G. Manning, Yanhui Zhang, Jing Jing Wang, Finn Purcell-Milton, Anuj Pokle, Stephen-Barry Porter, Chuan Zhong, Jing Li, Rudi O’Reilly Meehan, Ryan Enright, Yurii K. Gun’ko, Valeria Nicolosi, John J. Boland, Stefano Sanvito, John F. Donegan

**Affiliations:** 1 School of Physics, Trinity College Dublin, Dublin 2, Ireland; 2 School of Chemistry, Trinity College Dublin, Dublin 2, Ireland; 3 Centre for Research on Adaptive Nanostructures and Nanodevices (CRANN), Trinity College Dublin, Dublin 2, Ireland; 4 Advanced Materials and BioEngineering Research Centre (AMBER), Trinity College Dublin, Dublin 2, Ireland; 5 Thermal Management Research Group, Efficient Energy Transfer (ηET) Department, Nokia Bell Labs, Nokia, Dublin 15, Ireland

**Keywords:** Optics and photonics, Nanoscience and technology

## Abstract

Considerable attention has been drawn to the lead halide perovskites (LHPs) because of their outstanding optoelectronic characteristics. LHP nanosheets (NSs) grown from single crystalline lead halide possess advantages in device applications as they provide the possibility for control over morphology, composition, and crystallinity. Here, free-standing lead bromide (PbBr_2_) single-crystalline NSs with sizes up to one centimeter are synthesized from solution. These NSs can be converted to LHP while maintaining the NS morphology. We demonstrate that these perovskite NSs can be processed directly for fabrication of photodetector and laser arrays on a large scale. This strategy will allow high-yield synthesis of large-size perovskite NSs for functional devices in an integrated photonics platform.

## Introduction

Lead halide perovskites (LHPs) with the form APbX_3_ (A = inorganic or organic cation; X = halide anion) have attracted ever-increasing interest because of the high-efficiency solar cells which can be fabricated by solution methods. Since the pioneering work on high-performance LHP solar cells^1–3^, great effort has been made in developing these materials for solar cells, photodetectors, X-ray detectors, light-emitting diodes and lasers^4^. The strong optical response of the perovskites results from high absorption coefficients as well as long carrier diffusion lengths and high quantum efficiency in luminescence. Moreover, tunable wavelength laser emission can be realized by changing the stoichiometric ratio of halide in mixed halide perovskites, making LHPs not only efficient but an adaptable platform for applications^4–8^. It is possible to control the LHP film thickness and size for optoelectronic devices by using thermal evaporation, dip coating, and spin-casting. The LHP films fabricated by these methods are generally polycrystalline with significant roughness. Such loss of LHP film quality causes charge transport suppression by trap states with the resultant deterioration in their photophysical properties. For instance, a large amount of grain boundaries are exhibited in polycrystalline materials, in which traps reduce the photocarrier lifetime and quantum efficiency and ultimately lower device yield^9^.

To improve the electrical and optical properties of the thin films, it has become critical to understand and to finally achieve optimized crystals with large sizes and appropriate thicknesses^10^. Various strategies have been explored to synthesize high quality LHP single crystals such as anti-solvent assisted crystallization^11^, classical cooling method^12^, solution growth with a top-seed^13^, as well as inverse temperature crystallization^14^. Unfortunately, the one-step growth of large-size single-crystalline thin films remains challenging, partly due to the high-symmetry cubic lattice of the perovskites. These methods tend to form compact, small aspect ratios, high-symmetry crystals which needs further cutting or exfoliation for device applications^11,13,15^. Instead of the one-step direct growth of perovskite single crystals, an alternative way to fabricate large-size LHP nanosheets (NSs) is from single crystalline lead halide. Some groups have reported high-quality lead halide and LHP nanoplatelets synthesis by chemical vapor deposition (CVD) and physical vapor deposition (PVD) methods and studied the excellent optical properties related to different nanoplatelet thicknesses^16–19^. Nevertheless, the sizes of such platelets are less than 1 mm. Compared with the three-dimensional (3D) perovskite, two-dimensional (2D) perovskites which tend to form nanosheets inheriting from their layered structures, similar to the transition metal chalcogenide (TMD) materials are now an emerging field. For example, Dou *et al*. synthesized atomically thin 2D perovskite nanosheets from solution with the mixture of three different solvents, where the crystals grew on the substrate when the solvent evaporated^20^. Ma *et al*. report the solution synthesis of hundreds-of-nanometer thick 2D perovskites microplates through a recrystallization process^21^. In spite of these efforts, there is still no report on centimeter-size nanosheets made of 2D perovskite till now; and the control over perovskite synthesis is still in its infancy compared with other semiconductor NSs^22,23^. A highly controllable strategy to reproducibly synthesize perovskite NSs would allow scalable fabrication of high-performance perovskite devices^24^. Our goal here is developing a robust strategy that would grow centimeter-size lead halide NSs without using any large-scale or expensive equipment. In this study, we present a facile self-assembly approach for the synthesis of centimeter-scale free-standing (as opposed to surface-supported) PbBr_2_ NSs (≈1 cm in lateral size and ≈100 nm in thickness), using a low-cost process. A thermodynamic growth model offers guidelines for further improvement of stable fabrication of large NSs. Optical microscopy and scanning electron microscopy (SEM) imaging show the crystalline nature of the PbBr_2_ NS over a large area. These NSs can be converted to MAPbBr_3_ perovskite NSs by a gas-solid reaction. We have shown photodetection performance in these high quality MAPbBr_3_ NSs. Further, we demonstrated room-temperature lasing in an array based on these perovskite NSs. Demonstrations in both photodetector and laser arrays indicate the very strong potential of these perovskite NSs for device applications with high yield.

## Single-Crystal NS Growth and Structural Characterization

**Figure 1:**
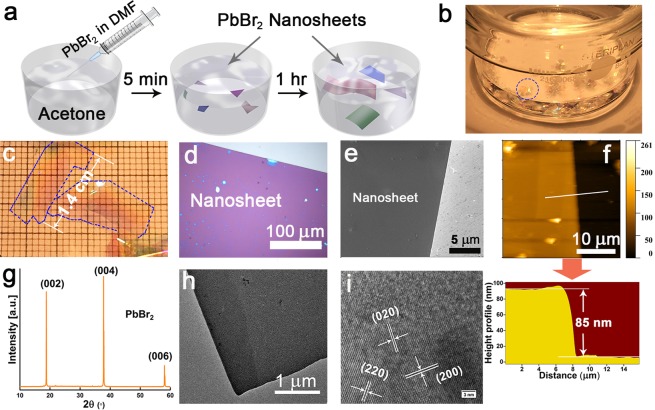
Characteristics of single-crystalline PbBr_2_ NSs. (**a**) Schematic illustration of the PbBr_2_ NSs preparation. (**b**,**c**) Images of the as-prepared PbBr_2_ NSs in solution and on TEM grid. (**d**) Optical image of an individual PbBr_2_ NS. (**e**) SEM images of PbBr_2_ NS. (**f**) AFM topography images of a single PbBr_2_ nanosheet with height profile underneath. (**g**) XRD pattern of PbBr_2_ ultrathin films. Diffraction peaks corresponding to 002, 004, 006 of the lead bromide crystals. (**h**) TEM image of PbBr_2_ NS. (**i**) High-resolution TEM image of a PbBr_2_ NSs.

In this work, PbBr_2_ NSs were synthesized by the reprecipitation method. In a typical preparation, 1 mL of a stock solution of PbBr_2_ (C = 0.1 M) in the good solvent of DMF was injected into 7 mL of the poor solvent acetone/ethanol at ∼ 40 °C, as illustrated in Fig. 1a. After around 1 hour, light-reflecting crystals were obtained as shown in the visible changes from Fig. 1b,c, with thickness of ∼100 nm and size up to ∼1.4 cm. The formation of PbBr_2_ NSs is spontaneous, notably, with no surfactant or catalyst in this synthesis. NSs were transferred to a substrate by inserting the substrate below the floating NS and slowly scooping up and washing with acetone. Chen *et al*. developed a space-limited inverse temperature crystallization (SLITC) technique to achieve the synthesis of submillimeter-size perovskite single crystal thin film, in which the key point is limiting the lateral growth of perovskite crystal in a confined space. However, it’s not easy to transfer the nanosheets to other substrate after the growth^25^. On the contrary, we are able to obtain large free-standing nanosheets in solution. These PbBr_2_ NSs are stable, they can be cast on diverse substrates including silicon, glass or quartz and can be stored for months without degradation, which is essential for device applications^26^.

Figures 1d,e and S1 present the optical and SEM images of the PbBr_2_ NSs drop-cast on a silicon substrate. Atomic force microscopy (AFM) images show that the thickness of the PbBr_2_ NS was ~ 85 nm, while the surface of PbBr_2_ NSs is smooth with ∼ 0.1 nm roughness (Figs 1f and S1). X-ray diffraction (XRD) was used to investigate the structure of PbBr_2_ NSs as shown in Fig. 1g. The characteristic peaks corresponding to the (002), (004), (006) planes of the lead bromide crystal (space group: *Pnam*(62), JCPDS file No. 31-0679). can be clearly observed The XRD pattern indicates that the crystals are well structured with only 3 diffraction peaks appearing, which further confirms that the obtained thin sheets are single crystals oriented along the [001] direction. As shown in Fig. 1h, transmission electron microscopy (TEM) was used to characterize the crystalline structure of the as-prepared PbBr_2_ NSs. The high-resolution TEM (HRTEM) result in Fig. 1i also demonstrates that the PbBr_2_ NS is single crystalline. Thus, the NSs in this work combine the lateral dimension of bulk crystals with nanometer-scale thickness. Namely, when such NSs grow laterally, their thickness remains essentially fixed. Moreover, because the NSs are prepared by direct self-assembly, they are immune to the damage typically caused in exfoliating the layered crystals. The bottom-up growth method developed here offers NSs with enlarged lateral sizes at high yield, compared to the exfoliation-based, top-down approaches. Moreover, their large aspect ratio (>10^5^) and thickness make these NSs advantageous for thin-film device applications. Further improvement of this strategy could result in the precise control of NS thickness and the fabrication of heterostructures for various electrical and optical device applications.

## Crystallization Model for Lead Bromide Single-Crystal NS Growth

**Figure 2:**
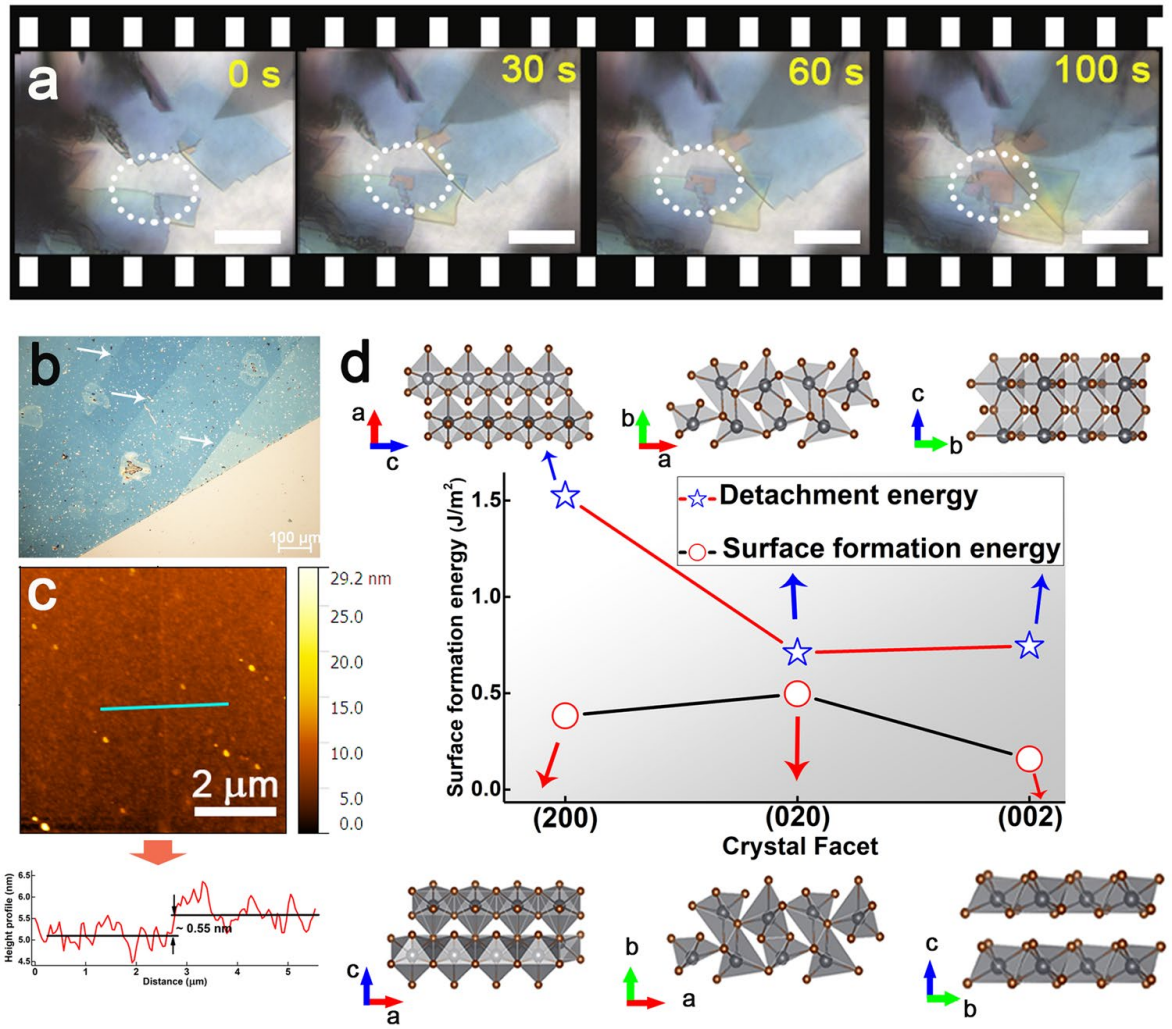
Growth process of NSs and surface energy calculation. (**a**) Growth evolution of crystal with time, which is also shown in Supplementary materials. (**b**) Image of a single NS with layered structure on it. (**c**) AFM image and height profile (underneath) of a monolayer. The thickness is around 0.5 nm (±0.1 nm). (**d**) The calculated detachment energies (*σ*_*d*_, J/m^2^) and the surface formation energies (*σ*_*f*_, J/m^2^) for the (200), (020) and (002) surfaces of PbBr_2_.

Using an optical microscope it is possible to study the two dimensional planar growth of NSs. It is expected that growth occurs by burst nucleation because the NSs become detectable after a very short time^27,28^. The nucleation phase is still unclear in our study limited by the resolution of the optical microscope^29^. Figure 2a shows the real-time growth evolution of NSs observed with an optical microscope, where we can observe the nucleation and growth of an individual NS identified by the white dotted circle. See video of growth in Supplementary Materials. The NS growth occurs along the two in-plane directions while the thickness does not significantly increase as there is no significant change in color of the NSs during the growth. In view of the driving force for two-dimensional crystallization, the most widely-accepted crystal growth mechanisms are spiral and layered growth^30^. As we did not observe spirals on the NSs, we believe that the growth follows the layered mechanism.

For PbBr_2_ NS growth, a new island will nucleate firstly on a facet of the sheet. When reaching a critical size, this island will then become stable and will acquire a driving force to expand, and finally a new adlayer will be added to the facet itself  ^31,32^. Inspection of the as-prepared NS reveals the presence of layered structures on the surface of the NS (Fig. 2b). These atomically thin NSs can only survive on top of the NSs, they tend to break or aggregate in the solution. The thinnest NS estimated from the AFM observations is determined to be 0.5 nm, which correspond to a monolayer (Fig. 2c). These top layers are likely to be generated by two-dimensional nucleation, which then spread and merge to cover the surface. The planar structure of the NSs demonstrates that the growth rate of out-of-plane is much lower than that in-plane due to the distinct surface energies in different directions. In general, the surface energy along the in-plane direction is expected to be lower than that out of plane, thus leading to a faster planar growth rate. The surface stability can be evaluated by assessing the following quantity:

$${\sigma }_{d}=\frac{1}{2A}({E}_{slab}-{E}_{bulk})$$

where *A* is a constant, *E*_*slab*_ and *E*_*bulk*_ are the total energy of a surface slab and of the corresponding bulk PbBr_2_, respectively. The so-defined detachment energy, *σ*_*d*_, was derived from the equation above by using density functional theory (DFT) as the total energy of a surface slab before structural relaxation (see Methods section). After structural relaxation the surface formation energy, *σ*_*f*_, should be used instead. This is calculated from the same equation by using the total energy of the fully-relaxed surface slab^33^.

Our results are illustrated in Table 1 and Fig. 2d ^34^. The detachment energy of the (020) surface appears to be the lowest, it is slightly higher than the (002) surface, while the (200) one has the highest detachment energy. However, after considering the structural relaxation, which enables the release of the surface stress, the (002) surface is found to be most stable so that it has the lowest surface formation energy, followed by the (200) and (020) surface. The insets of Fig. 2d illustrates the structural relaxation of (002) surface, with no structural relaxation being observed for the (200) and (002) surfaces. According to the Curie-Wulff relation, at equilibrium between the island and the solution one has *σ*_*1*_/*l*_*1*_ = *σ*_*2*_/*l*_*2*_ = *g*, where *σ* is surface formation energy, *l* is the length in the respective crystallographic directions and *g* is a constant. It follows that if *σ*_*1*_ > *σ*_*2*_, then *l*_*1*_ > *l*_*2*_, namely the island grows elongated along the direction of higher surface free energy. This minimizes the area of facets with higher *σ*. The (002) surface is stabilized by the unique structural relaxation and its low energy state ensures its energetic preference and suppresses the growth of PbBr_2_ NSs along the [001] direction.

**Table 1:** Surface detachment (*σ*_*d*_) and formation (*σ*_*f*_) energies of different crystal facets.

Surface energy (J/m^2^)	(200)	(020)	(002)
σ_d_	1.524	0.710	0.745
σ_f_	0.383	0.497	0.159

## Conversion of Lead Bromide to Perovskite

**Figure 3:**
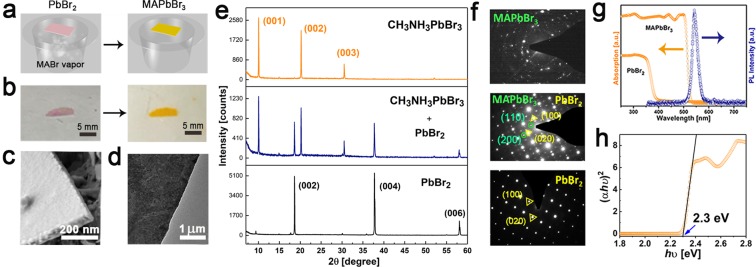
Conversion of PbBr_2_ to MAPbBr_3_. (**a**) Schematic illustration and (**b**) Images of the vapor-phase conversion process to transform PbBr_2_ into a NS MAPbBr_3_ perovskite. (**c**) SEM and (**d**) TEM images of MAPbBr_3_ NSs. (**e**) XRD pattern of MAPbBr_3_ NSs. (**f**) Corresponding electron diffraction pattern of MAPbBr_3_ NS. (**g**) Steady-state photoluminescence and absorption spectra of PbBr_2_ and perovskite materials. (**h**) Corresponding Tauc plots displaying the extrapolated optical band gaps of MAPbBr_3_ NSs.

The prepared PbBr_2_ NSs can be converted to methylammonium (MA) lead bromide perovskites by a vapor-solid reaction in MABr vapor. Figure 3a,b demonstrates the conversion of the purple colored PbBr_2_ to the orange-colored MAPbBr_3_, which can be completed after 80 hours. The planar structure is maintained in the resulting MAPbBr_3_ perovskite, showing good retention of morphology after conversion^32^. Comparing to the conversion in solution, the vapor-solid reaction can retain the morphology and crystallinity within the perovskite crystals while preventing the dissolution of PbBr_2_ and perovskite crystals in organic solvents^7,35,36^. After conversion, some NSs become slightly roughened, as observed in SEM (Fig. 3c) and TEM images (Fig. 3d), due to strain experienced during the expansion. The XRD pattern can be fully indexed to the orthorhombic phase of MAPbBr_3_, indicating the complete conversion from PbBr_2_ (Fig. 3e). It has been reported that the crystal structure of MAPbI_3_ perovskite synthesized by vapor-solid conversion method is a tetrahedral structure, while the solution-grown MAPbI_3_ crystal structure has a cubic structure at room temperature^24,37^. Similarly in our case, by vapor-solid conversion from PbBr_2_, we obtain orthorhombic phase MAPbBr_3_ nanosheets rather than the cubic phase MAPbBr_3_ crystals which can be synthesized from solution^38^. In order to confirm the crystal structure of the perovskite NSs, electron diffraction (Fig. 3f) was used to establish the integrity of the crystal lattice by revealing the well-defined Bragg reflections. The electron diffraction pattern shows that oriented highly crystalline domains are formed after conversion to LHP, these perovskite NS are very likely polycrystalline when we compare with vapor phase conversion of PbX_2_^17,24,37,39^. The steady-state photoluminescence (PL) and absorption of the perovskites are investigated in Fig. 3g. The peak of the PL is close to the band edge (545 nm)^39^. The absorption spectrum indicates that the band edge is at 539 nm (2.3 eV, Fig. 3h). A low concentration of mid-gap defects is suggested by the steep rise in absorption at the band edge.

## Photodetector Arrays Based on Perovskite NSs

**Figure 4:**
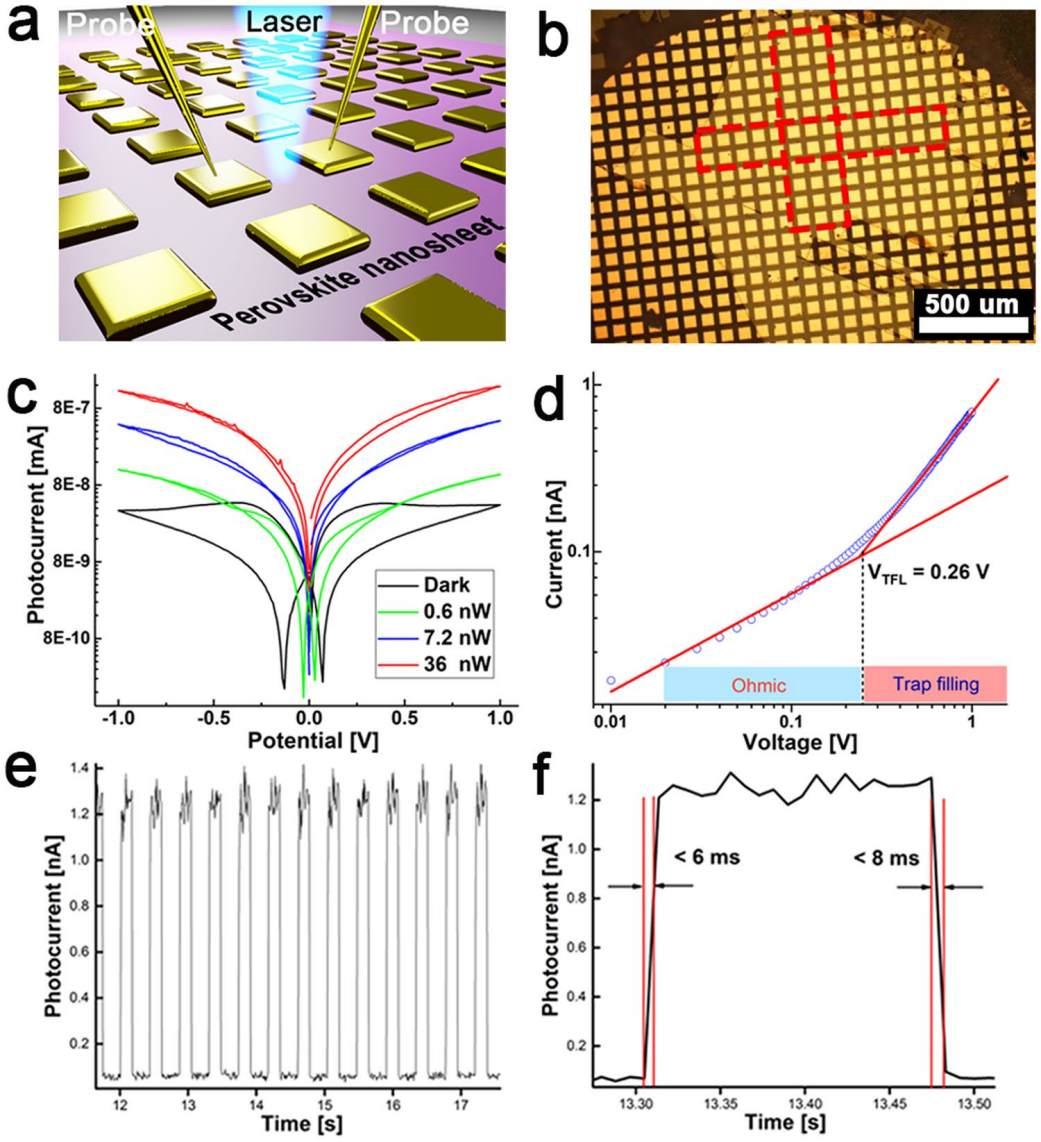
Photodetector measurements. (**a**) Schematic representation of the experimental setup for photodetector measurement, the laser spot was focused between the two electrodes. (**b**) Optical images of the device arrays on glass substrate. (**c**) *I-V* curves of the NSs perovskite-based device under laser irradiation with different powers. (**d**) Current-voltage trace of perovskite NSs. It shows a linear ohmic region followed by the trap-filled region at *V*_*TFL*_ = 0.26 V. The trap density determined by *V*_*TFL*_ was calculated to be *n*_*trap*_ = 1.17 × 10^10^ cm^−3^. (**e**) Time-dependent photocurrent measurement on the perovskite photodetector under the different power of a 405 nm laser with a voltage bias of 1 V. Time-resolved photoresponse. (**f**) Transient photocurrent of the photodetector (bias, 1 V; λ = 405 nm). The rise time (<6 ms) is defined as the time for the photocurrent to increase to 70% of the ON-state current. The fall time (<8 ms) is defined as the time for the photocurrent to decrease by 70% of the ON-state current.

One potential application of these materials is their use as ultrathin media for the fabrication of photodetectors. A schematic representation of the experimental setup is shown in Fig. 4a. The large size of the crystals allows us to directly deposit patterned electrodes on perovskite NSs to form functional devices with a patterned shadow mask (e.g. copper grid in this case). In this way, we can prepare large area independently addressable devices by selectively depositing an array of electrodes on the perovskite NSs (Fig. 4b) without the need for any advanced lithographic processes^24^. A continuous wave (CW) laser (405 nm) was focused on the channel of each device by an objective, with the diameter of laser spot of around 3 μm.

Figure 4c shows the typical current-voltage (*I-V*) curves of the NSs-based device under laser illumination with different powers. Electrical measurements of these devices exhibit a nearly linear *I-V* behavior under irradiation and a low dark current, showing a strong response of the LHP crystals to light. When a weak laser (405 nm, effective power < 10 nW) was irradiated on the NS-based device, the *I-V* curves of the device show hysteresis, namely the current is dependent on the sweeping direction of voltage between the two electrodes. This feature is attributed to some residual poling effect in MAPbBr_3_^40^. The ratio of the photocurrent to the dark current can reach up to 2 orders of magnitude, which is comparable with other work. According to the relation *R*_*λ*_ = *I*_*ph*_/*PS*, where *I*_*ph*_ is the photocurrent; *P* is the light power intensity irradiated on the device; and *S* is the effective area of the photodetector, we obtain a photoresponsivity of ≈ 0.14 AW^−1^ under 405 nm incident light with a power intensity of 1.3 mW · cm^−2^ at 1 V bias. We can estimate the trap density of the LHP NSs by using the space charge-limited current (SCLC) technique^25^. As exhibited in Fig. 4d, the *I-V* curves at low bias exhibit an Ohmic response; on the contrary, the traps are filled with the nonlinear rise of the current at high bias, indicating the trap-filled limit (TFL) regime^14^. The trap density determined by *V*_*TFL*_ was calculated to be 1.17 × 10^10^ cm^−3^, which is comparable with that reported for bulk perovskite single crystals. The time-resolved photocurrent of the photodetector shows high photo-switching performance (Fig. 4e,f) limited by the experimental setup. In this work, we have fabricated a large array of photodetectors on glass substrate and selected devices across the area along two directions to test the photocurrent (marked by red boxes in Fig. 4b). More than 50 devices have been measured in this work and all of them show strong photoresponse under laser irradiation.

## Laser Arrays Based on Perovskite NSs

**Figure 5:**
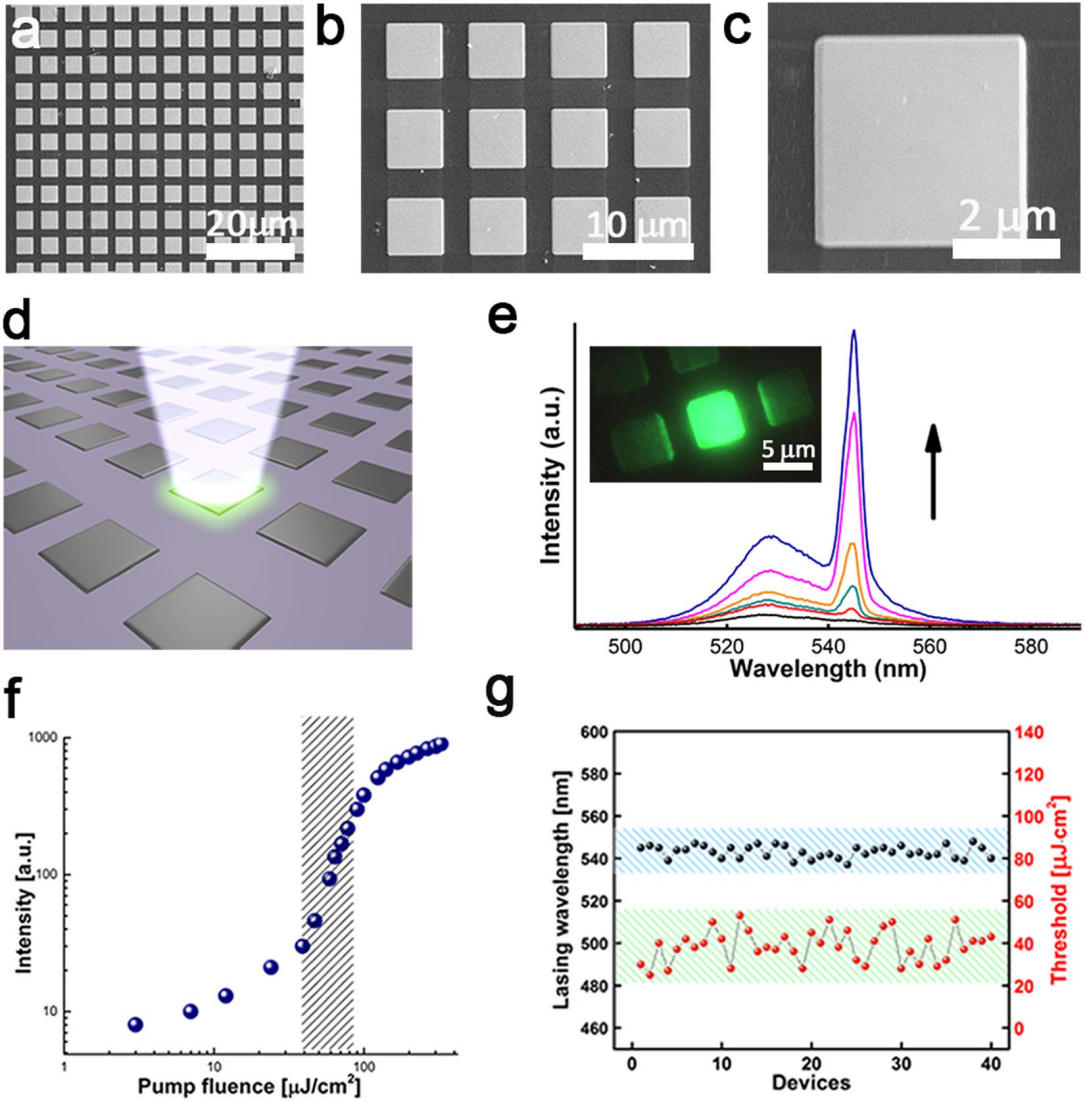
Room-temperature lasing characteristics. (**a**–**c**) SEM micrograph of the microcavity arrays. Low-magnification SEM images of focused ion-beam milled section. (**d**) Schematic representation of the excitation method. (**e**) Spectra at six different optical pump fluences, showing the transition from spontaneous emission to amplified spontaneous emission and to lasing. Inset: Optical images showing emission from the microdisk. (**f**) Nonlinear response of laser output power with increasing pump fluence, showing threshold region as a ‘kink’ between the two linear regimes of spontaneous emission and lasing. The shadow area is the region of amplified spontaneous emission. (**g**) Variation in wavelength and threshold for 40 measured devices.

Perovskites can provide sufficient gain for lasing at room temperature^41,42^. As illustrated in Fig. 5a–c, uniform arrays of PbBr_2_ microcavities (μ-cavity) were fabricated on silicon substrates using focused ion beam (FIB) lithography techniques yielding more than one hundred μ-cavities in an area of 80 μm × 80 μm. These PbBr_2_ μ-cavities can be converted to MAPbBr_3_ μ-cavities by the gas-solid reaction. Room-temperature (RT) laser oscillation is achieved by optical pumping with a femtosecond (fs) laser. Figure 5d presents a schematic of a μ-cavity on a silicon substrate, which is optically excited using a 400 nm fs laser, illuminating an individual μ-cavity. Figure 5e shows the MAPbBr_3_ μ-cavity emission spectra and the inset shows the optical images under the laser illumination. At low pumping energy levels (<45 μJ/cm^2^), the MAPbBr_3_ μ-cavity emits with a broad spectral peak. At high pumping energy levels, a cavity mode appears, and laser oscillation emerges at ~545 nm. The light-input–light-output power relationship, or “*L-L* curve” is shown as log-log plot in Fig. 5f. Wavelength and threshold variation of 50 different laser devices are shown in Fig. 5g. All the lasers characterized have similar emission wavelengths around 540 nm.

We have demonstrated a facile reprecipitation method for scalable growth of lead bromide single crystalline NSs with centimeter lateral size and nanoscale thickness. The method we used to grow the NSs is scalable, does not require complex equipment, and provides freestanding NSs in solution, which can be deposited onto any substrates. We have then described the growth mechanism of single-crystalline PbBr_2_ NSs and demonstrated that the as-prepared PbBr_2_ NSs can be converted to lead halide perovskite materials such as MAPbBr_3_. Our methodology facilitated the growth of ultrathin lead bromide films that do not naturally present themselves as layered systems, providing a synthetic pathway toward a different class of large-size and ultrathin perovskite materials that were previously inaccessible from one-step crystal growth. The demonstrated excellent optical and photoelectric properties of these converted MAPbBr_3_ NSs make them a potential candidate for nanolasers and photodetectors. The arrays of lasers and photodetectors all worked as would be expected for a high-yield of devices from high-quality materials. The laser thresholds and photodetector efficiencies do not yet meet the state-of-the-art, due to the simple nature of the device geometries used, but will be improved in the future. Beyond our results described above, a step closer to the wafer-scale devices can be expected by increasing the crystal size, further improving the crystallinity, and extending to other metal halide materials.

## Materials and Methods

### Reagents

Unless otherwise stated, all of the chemicals were purchased from Sigma-Aldrich Chemical and used as received.

### Synthesis of lead bromide (PbBr_2_) nanosheets

The PbBr_2_ (734 mg, 2 mmol) were dissolved in 20 mL of anhydrous *N*, *N*’-dimethylformide (DMF) inside a 25 mL vial. Then, 1 mL of a stock solution of PbBr_2_ (C = 0.1 M) in the good solvent of DMF was injected slowly into the poor solvent of 5 mL/2 mL acetone/ethanol at 40 °C. The mixture was kept at this temperature without disturbance for 1 hour. A light-reflecting crystal suspension was obtained as shown in the visible changes from Fig. 1b, with thickness of ∼100 nm and size up to ∼1 cm. The thickness of the NSs can be tuned from 0.5 nm (monolayer) to 1 μm, depending on time of growth, but not yet in a controllable fashion. After the growth, NSs were transferred to a substrate by slowly scooping up the floating NSs, washing with acetone and repeated for three times. After washing, the sample was dried at 60 °C in a vacuum oven for 2 h. Formation of PbBr_2_ NSs is spontaneous, notably, with no surfactant or catalyst in this synthesis. The product does not need any further purification. See Supplementary Fig. S1 also. Attempts to make NSs of PbCl_2_ and PbI_2_ with this method were not successful, nanowires were formed instead.

### Conversion of lead bromide (PbBr_2_) to perovskite NSs

After obtaining reproducible and high-quality solution-grown PbBr_2_ NSs, we converted them to MAPbBr_3_ perovskite NSs via a vapor-phase reaction. Conversion by vapor-phase methylammonium bromide (MABr) was performed as illustrated in Fig. 2a. Within a glove box, a 20 mL glass vial was loaded with 0.1 g of CH_3_NH_3_Br. Afterwards, a substrate containing PbBr_2_ NSs was placed in the vial face up. The vial was carefully sealed with a rubber septum and held in place using a cable tie, and the vial was placed half submerged in an oil bath at 140–150 °C. Alternatively, a capped vial heated in a glove box with a hot plate set to 160 °C could also be used. After several hours, CH_3_NH_3_Br deposition was apparent on the sidewalls of the vial. The reaction was allowed to proceed until the film was uniformly orange, which required up to 80 hours for full conversion^43^. Partial anion exchange could be achieved by controlling reaction time. The resulting MAPbBr_3_ perovskite still maintains the planar structure, indicating good morphology retention after vapor-phase conversion.

### **DFT results**

Density functional theory (DFT) calculations have been performed by using the all-electron code FHI-AIMS^33^. A pre-constructed high-accuracy all-electron basis set of numerical atomic orbitals was employed, as provided by the FHI-AIMS “tight” option. The exchange-correlation energy was described by the Perdew-Burke-Ernzerhof (PBE) generalized gradient approximation (GGA)^44^. Long-range van der Waals interactions have been taken into account with the Tkatchenko and Scheffler (TS) DFT scheme^45^. Relativistic effects associated to the Pb heavy metal were treated by using the atomic ZORA approximation as described in ref. ^45^. The atomic structures of the PbBr_2_ surfaces were constructed by cleaving optimized bulk PbBr_2_ along the [200], [020] and [002] directions. Supercells with 2 × 1 × 2, 1 × 2 × 2 and 1 × 1 × 4 shape were adopted for (200), (020) and (002) surface slabs, respectively, giving a surface area of 76.92, 90.25 and 76.37 Å^2^. These surface areas are large enough to allow surface reconstruction. Moreover, a vacuum region of 16 Å was also added beside the cleaved slabs to avoid the spurious interaction between adjacent periodic cell replica. A dense *k*-mesh of size 1 × 12 × 11, 11 × 1 × 11 and 11 × 12 × 1 was used to sample the reciprocal space integration of the (200), (020) and (002) surface slabs, respectively. A process of structural optimization to release the internal stresses (i.e. the surface relaxation) was performed for all the surface slabs. Here, the structural optimization was performed with the Broyden-Fletcher-Goldfarb-Shanno algorithm, with a force tolerance of 0.05 GPa. The out-of-plane structural parameters (both the lattice size and the atomic positions) were further optimized. At the same time the in-plane ones was kept fixed to the bulk PbBr_2_ value.

## Supplementary information

Supplementary Information File

Supplementary Movie PbBr_2_ Nanosheet Growth

## Data Availability

All data needed is provided in the paper and the supplementary materials to evaluate the conclusions of our work.
