# Supplementary material for: Synthesis of centimeter-size free-standing perovskite nanosheets from single-crystal lead bromide for optoelectronic devices
Source: Sci Rep. 2019 Aug 13;9:11738. doi: 10.1038/s41598-019-47902-1 (PMC6692359; doi:10.1038/s41598-019-47902-1)
Supplement: Supplementary file 1 — Supplementary Information File [file 41598_2019_47902_MOESM1_ESM.docx]

Supplementary Materials for

Synthesis of centimeter-size free-standing perovskite nanosheets from single-crystal lead bromide for optoelectronic devices

Jian-Yao Zheng,^1,3,4^ Hugh Manning,^2,3,4^ Yanhui Zhang,^1,3,4^ Jing Jing Wang,^3,4^ Finn Purcell-Milton,^2,3,4^ Anuj Pokle,^1,3,4^ Stephen-Barry Porter,^1,3,4^ Chuan Zhong,^1,3,4^ Jing Li,^2,3,4^ Rudi O'Reilly Meehan,^5^ Ryan Enright,^5^ Yurii K. Gun’ko,^2,3,4^ Valeria Nicolosi,^1,2,3,4^ John J. Boland,^2,3,4^ Stefano Sanvito^1,3,4^ and John F. Donegan^1,3,4,*^

**Fig. S1.** **Samples of PbBr_2_ NS**s. Top Row: Optical images showing crystals of several mm in lateral size on glass substrates. Scale bar refers this row only. Middle Row: AFM images and Lower Row: AFM Height Profile showing NSs thickness.


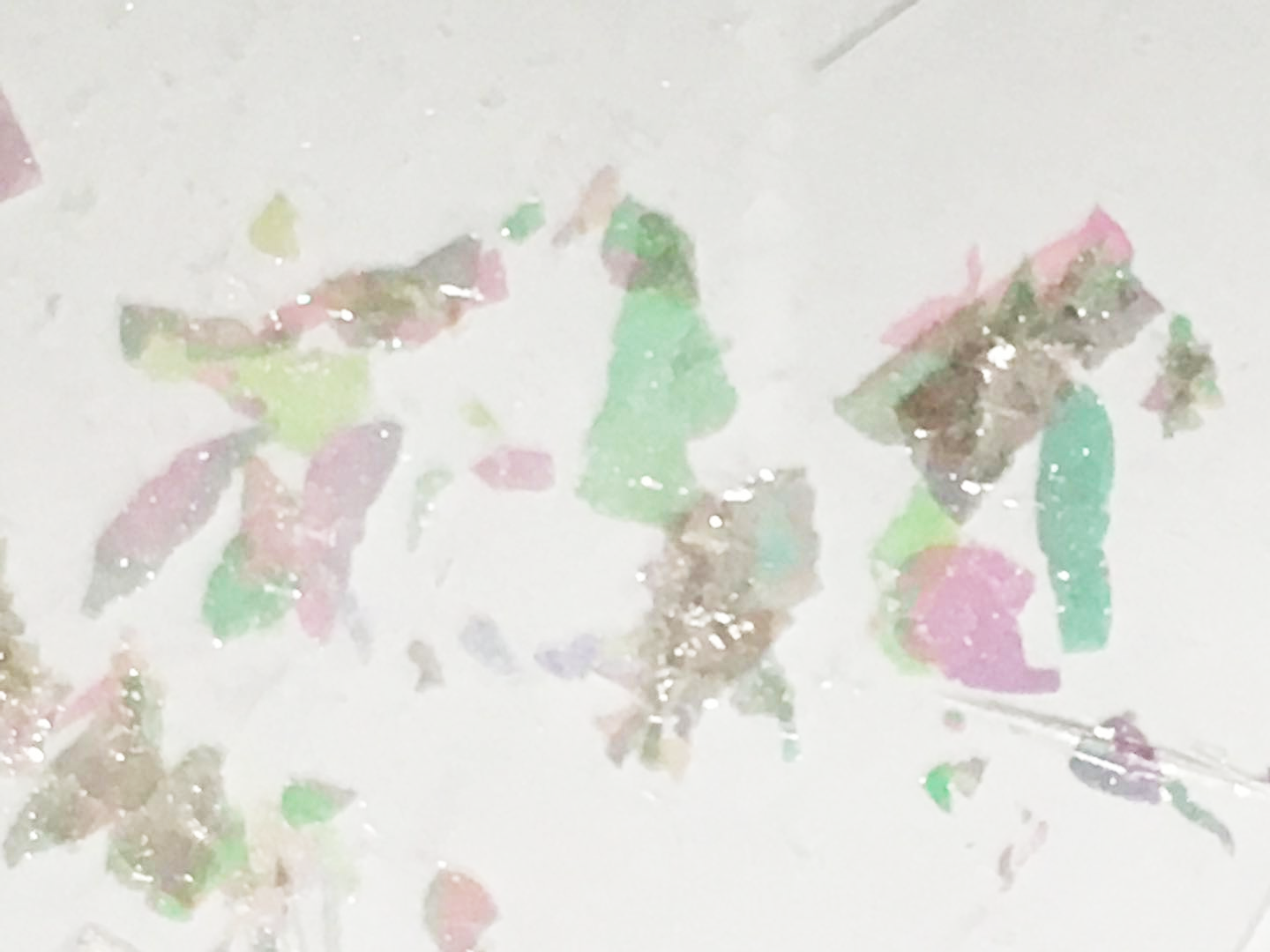

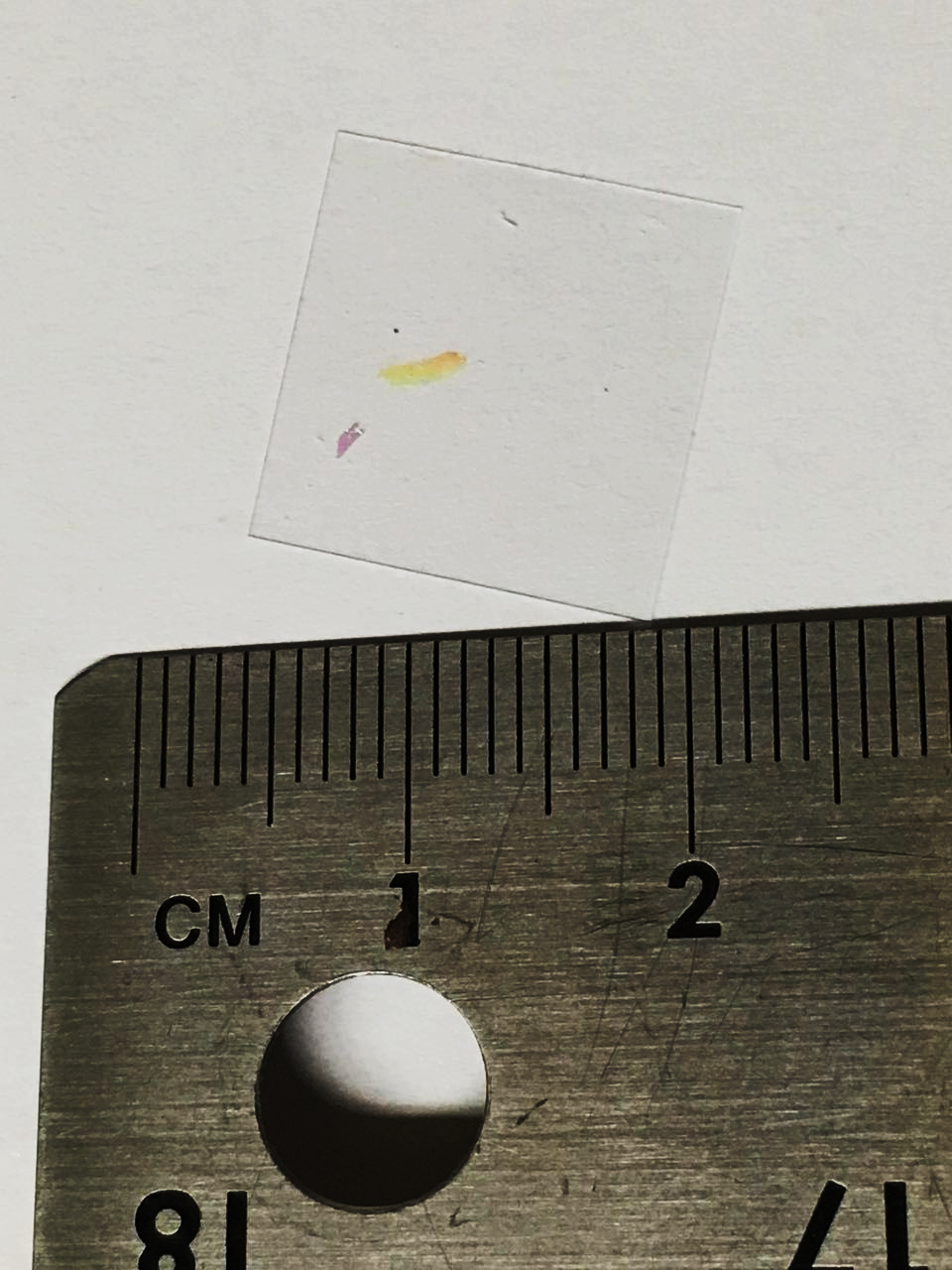

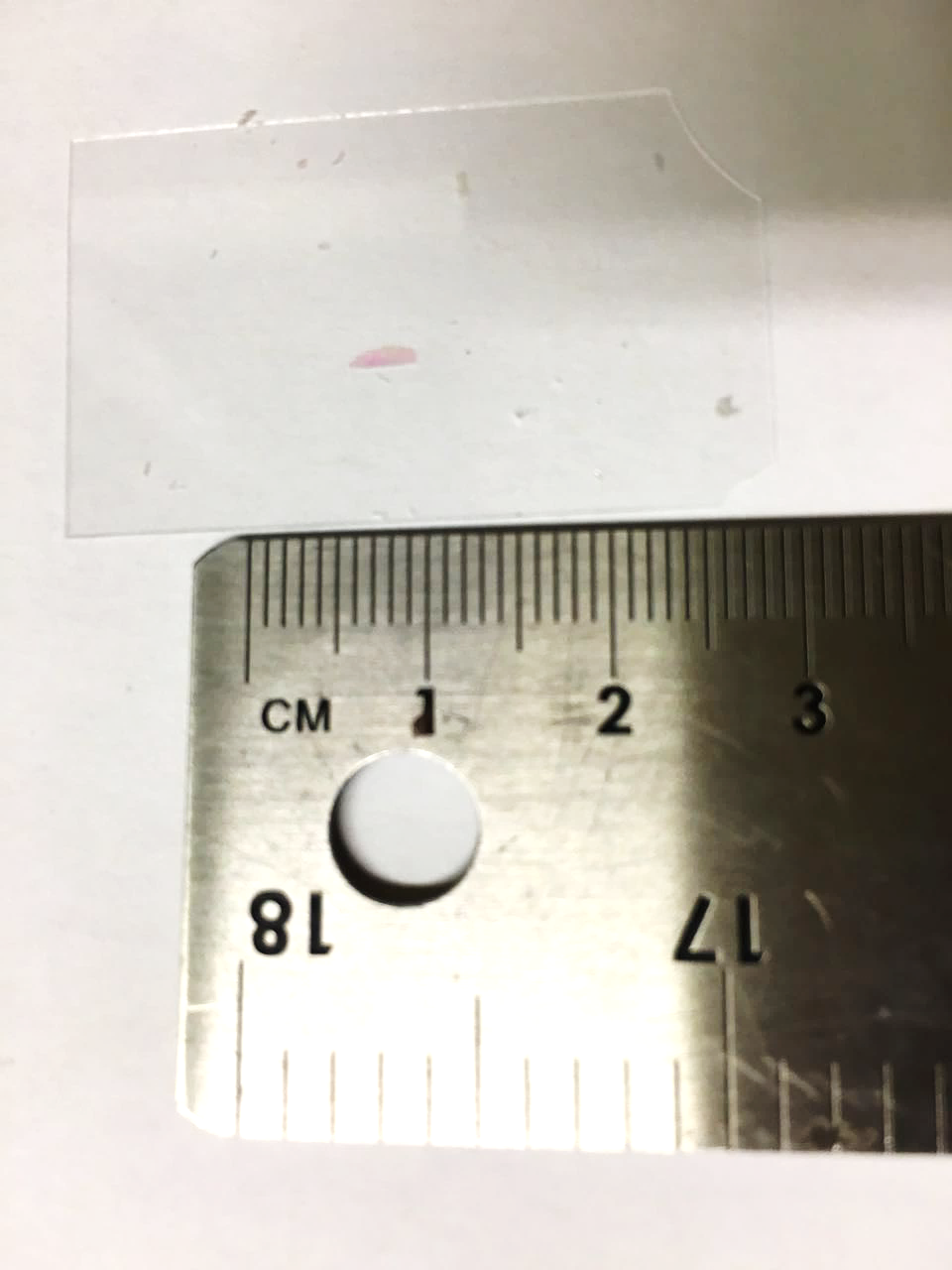

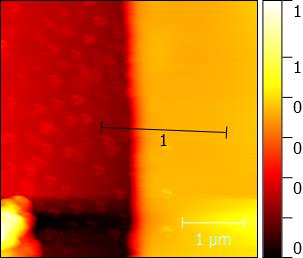

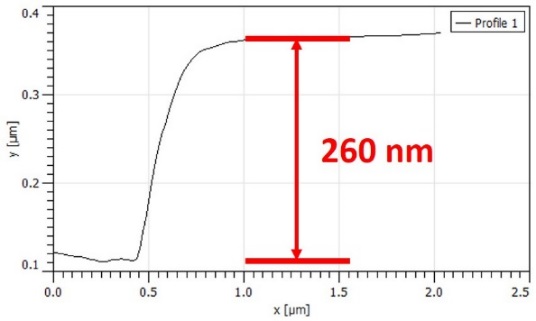

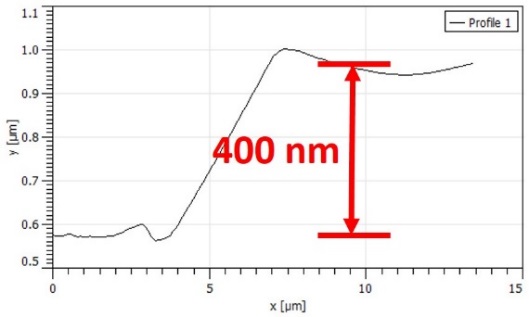

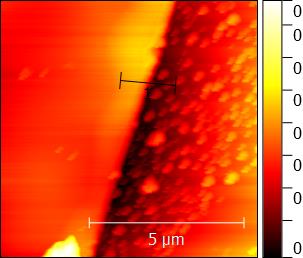

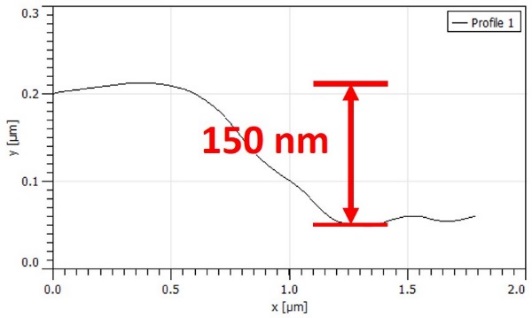

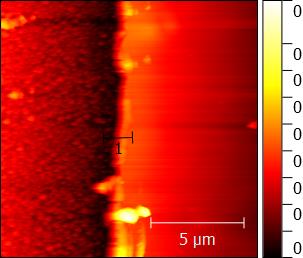


**5 mm**
